# NK cell modulation by JAK inhibition

**DOI:** 10.18632/oncoscience.224

**Published:** 2015-08-31

**Authors:** Kathrin Schönberg, Janna Rudolph, Dominik Wolf

**Affiliations:** Department of Internal Medicine III, Oncology, Hematology and Rheumatology, University Clinic Bonn (UKB), Bonn, Germany

**Keywords:** NK cells, MPN, JAK Inhibitor, myelofibrosis

NK cells are innate immune effector cells recognizing and killing virus-infected or malignant cells [1]. NK cell function is tightly regulated by a complex balance between various activating and inhibitory NK cell receptors. In addition, several cytokines (IL-2, IL-12, IL-15 and IL-18) mediated via the JAK/STAT pathway are critical for NK cell development and activation. Thus it is tempting to speculate that the novel class of immune-modulatory compounds (i.e. the JAK inhibitors) are able to modify NK cell biology *in vitro* and *in vivo*. We recently support this idea by demonstrating that the first approved JAK1/2/(3) inhibitor ruxolitinib dramatically impacts NK cell biology *in vivo* in patients suffering from myeleloproliferative neoplasia (MPN)[2]. Ruxolitinib-treated MPN patients have drastically reduced circulating NK cell numbers. Individuals deficient in NK cells due to an inherited genetic disposition (i.e. *JAK3* and/or *STAT5B* mutations that cause severe combined immunodeficiency (SCID) syndrome in humans) are prone to severe infections. Therefore, it is not surprising to see that patients with MPN exposed to the JAK inhibitor, who develop infections, have lower NK cell counts compared to non-infected individuals. However, we also clearly state that severe infections (tuberculosis, progressive multifocal encephalopathy, toxoplasmosis), even though described during ruxolitinib therapy, are relatively infrequent[3]. The most prevalent types of infections during ruxolitinib therapy are urinary tract infection and herpes zoster reactivation, which is known to be linked to NK cell deficiency.

However, there are huge differences between patients with inherited disruption of the JAK/STAT pathway as opposed to patients treated with ruxolitinib. Firstly, we could show that the drug-related effect is reversible. In patients who had to stop the therapy due to side effects, NK cell levels rose back to normal values. Furthermore, the main difference is that in patients exposed to the drug, JAK/STAT inhibition lasts only for several hours (3–4) due to the pharmacokinetic properties of the drug. As a consequence of the bi-daily intake of the drug, the total number of JAK/STAT inhibition may last a maximum of 6–8 hours[4]. In between the JAK/STAT pathway is active and allows residual cells to be activated relatively normal. In contrast, for limiting the hyper-inflammation going along with the MPN, this inhibition time appears to be sufficient to drastically reduce symptom burden and shrink the spleen[5]. Furthermore, it also suffices to clearly deplete NK cells in MPN patients. But why is this the case? The increased ratio of immature to mature NK cells during ruxolitinib therapy provides some hints, that the development of mature NK cells is affected, which could be explained by the inhibition of various important cytokine signals essential for NK maturation (i.e. IL-2 and IL-15). Reduced NK cell numbers in ruxolitinib-exposed patients may at least in part be due to defective NK cell maturation, which results in the time-dependent NK cell drop. In line with this idea, data demonstrated that NK cell precursors lacking IL-15Rβ also exhibit a NK cell differentiation defect leading to severe immunodeficiency. Moreover, IL-15R integrates signals *via* its common γ-chain in committed NK cell precursors and fosters immature to mature NK cell differentiation as well as the cytokine supports mature NK cell survival. Thus, it is tempting to speculate that ruxolitinib interferes with essential cytokine signals for terminal NK cells maturation and explaining the shift to the more immature NK cell phenotype.

Strikingly, ruxolitinib not only affects NK cell biology *in vivo*, but also impairs NK cell function *in vitro*. Mainly, cytokine-mediated activation is blocked by the drug and most importantly it inhibits NK killing activity, as it interferes amongst others also with the lytic synapse formation process. We are currently investigating in detail the impact of the drug on the migration process of immune cells and early data clearly highlight the effects of the compound on cytoskeletal rearrangement. This may at least in part also affect the potential of NK cells to go into stable contact with target cells, thereby limiting their killing activity. These data may be of particular relevance when considering the application of the drug as cancer-inflammation reverting compound (e.g. in pancreatic cancer[6]) or when applying the drug as immune-suppressant after allogeneic stem cell transplantation[7], as it may also interfere with cancer immune-surveillance or the graft-versus-disease effect in patients thereby fostering disease recurrence. Considering our data, an important question that arises is how long-term ruxolitinib therapy shapes the immune system after years or even decades. The figure depicts the major effects of the JAK inhibitor ruxolitinib on NK cells *in vivo* (in MPN patients) and *in vitro*.

**Figure 1:**
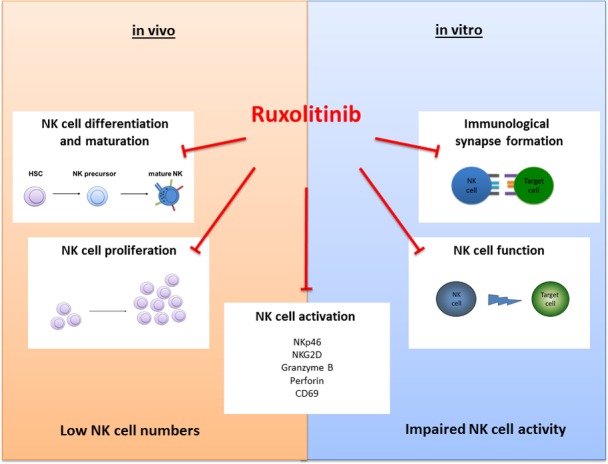
The figure depicts the major effects of the JAK inhibitor ruxolitinib on NK cells *in vivo* (in MPN patients) and *in vitro*
